# Oxidative DNA damage preventive activity and antioxidant potential of plants used in Unani system of medicine

**DOI:** 10.1186/1472-6882-10-77

**Published:** 2010-12-16

**Authors:** Mehar Darukhshan Kalim, Dipto Bhattacharyya, Anindita Banerjee, Sharmila Chattopadhyay

**Affiliations:** 1 Plant Biotechnology Laboratory, Drug Development/Diagnostics & Biotechnology Division, Indian Institute of Chemical Biology (A unit of Council of Scientific & Industrial Research), 4, Raja S.C. Mullick Road, Kolkata 700 032, India

## Abstract

**Background:**

There is increasing recognition that many of today's diseases are due to the "oxidative stress" that results from an imbalance between the formation and neutralization of reactive molecules such as reactive oxygen species (ROS) and reactive nitrogen species (RNS), which can be removed with antioxidants. The main objective of the present study was to evaluate the antioxidant activity of plants routinely used in the Unani system of medicine. Several plants were screened for radical scavenging activity, and the ten that showed promising results were selected for further evaluation.

**Methods:**

Methanol (50%) extracts were prepared from ten Unani plants, namely *Cleome icosandra, Rosa damascena, Cyperus scariosus, Gardenia gummifera, Abies pindrow, Valeriana wallichii, Holarrhena antidysenterica, Anacyclus pyrethrum, Asphodelus tenuifolius *and *Cyperus scariosus*, and were used to determine their total phenolic, flavonoid and ascorbic acid contents, in vitro scavenging of DPPH^·^, ABTS^·+^, NO, ^·^OH, O_2_^.- ^and ONOO^-^, and capacity to prevent oxidative DNA damage. Cytotoxic activity was also determined against the U937 cell line.

**Results:**

IC_50 _values for scavenging DPPH^·^, ABTS^·+^, NO, ^·^OH, O_2_^.- ^and ONOO^- ^were in the ranges 0.007 ± 0.0001 - 2.006 ± 0.002 mg/ml, 2.54 ± 0.04 - 156.94 ± 5.28 μg/ml, 152.23 ± 3.51 - 286.59 ± 3.89 μg/ml, 18.23 ± 0.03 - 50.13 ± 0.04 μg/ml, 28.85 ± 0.23 - 537.87 ± 93 μg/ml and 0.532 ± 0.015 - 3.39 ± 0.032 mg/ml, respectively. The total phenolic, flavonoid and ascorbic acid contents were in the ranges 62.89 ± 0.43 - 166.13 ± 0.56 mg gallic acid equivalent (GAE)/g extract, 38.89 ± 0.52 - 172.23 ± 0.08 mg quercetin equivalent (QEE)/g extract and 0.14 ± 0.09 - 0.98 ± 0.21 mg AA/g extract. The activities of the different plant extracts against oxidative DNA damage were in the range 0.13-1.60 μg/ml. Of the ten selected plant extracts studied here, seven - *C. icosandra, R. damascena, C. scariosus, G. gummifera, A. pindrow, V. wallichii *and *H. antidysenterica - *showed moderate antioxidant activity. Finally, potentially significant oxidative DNA damage preventive activity and antioxidant activity were noted in three plant extracts: *C. icosandra, R. damascena *and *C. scariosus*. These three plant extracts showed no cytotoxic activity against U937 cells.

**Conclusions:**

The 50% methanolic extracts obtained from different plant parts contained significant amounts of polyphenols with superior antioxidant activity as evidenced by the scavenging of DPPH^·^, ABTS^·+^, NO, ^·^OH, O_2_^.- ^and ONOO^-^. *C. icosandra, R. damascena *and *C. scariosus *showed significant potential for preventing oxidative DNA damage and radical scavenging activity, and the *G. gummifera, A. pindrow, V. wallichii, H. antidysenterica, A. pyrethrum, A. tenuifolius *and *O. mascula *extracts showed moderate activity. The extracts of *C. icosandra, R. damascena *and *C. scariosus *showed no cytotoxicity against U937 cells. In conclusion, these routinely used Unani plants, especially *C. icosandra, R. damascena *and *C. scariosus*, which are reported to have significant activity against several human ailments, could be exploited as potential sources of natural antioxidants for plant-based pharmaceutical industries.

## Background

The World Health Organization estimates that 80% of the world's inhabitants rely mainly on traditional medicines for their health care [1]. Herbs contain some of the most powerful natural antioxidants and are highly prized for their antioxidant and anti-ageing effects.

Natural products offer an untold diversity of chemical structures. These compounds often serve as lead molecules, the activities of which can be enhanced by chemical manipulation and by de novo synthesis [2,3]. To date, many medicinal plants have proved successful in combating various ailments, leading to mass screening for their therapeutic components.

Antioxidants are widely used as ingredients in dietary supplements and are exploited to maintain health and prevent oxidative stress-mediated diseases such as cancer, atherosclerosis, diabetes, inflammation and ageing. Recently, many antioxidants have been isolated from different plant materials [4-6]. Natural antioxidants are also in high demand for application as nutraceuticals and as food additives because of consumer preferences [7,8]. In addition to their uses in medicine, these compounds are used in industry e.g. as preservatives in food and cosmetics and for preventing the degradation of rubber and gasoline. Antioxidants are also used as additives to help guard against food deterioration. Among natural antioxidants, plant polyphenols+ are especially important [9]. Today, the search for natural compounds rich in antioxidant, anticancer and antimicrobial properties is escalating because of their importance in controlling many chronic disorders such as cancer and cardiovascular diseases [5]. It has been estimated that approximately two-thirds of anticancer drugs approved worldwide up to 1994 were derived from plant sources [10].

It is increasingly being realized that many of today's diseases are due to the "oxidative stress" that results from an imbalance between the formation and neutralization of prooxidants [6]. These excess free radicals react with biological macromolecules such as proteins, lipids and DNA in healthy human cells and this results in the induction of carcinogenesis, atherosclerosis, cardiovascular diseases, ageing and inflammatory diseases [11,12]. These harmful radicals have to be eliminated from biological systems by enzymes such as superoxide dismutase, catalase and peroxidase, or compounds such as ascorbic acid, tocopherol and glutathione, which possess antioxidant properties.

Unani medicine, a form of traditional medicine widely practiced in India and the rest of the Indian subcontinent, is orientated towards prevention, health maintenance and treatment. Herbal products are regularly used in traditional medicines such as Ayurveda and Unani, which strengthen body defences [13]. Unani therapies cure the diseases without such side effects even after they have been consumed for a long time with a wide spectrum of therapeutic activity. Unani therapies are known to be relatively economic and are most popular amongst people because they are safe and have time-tested efficacy. They contain vitamins, minerals, active steroids, alkaloids, glycosides and tannins as well as a variety of antioxidants in a biologically natural state.

In this study we screened several Unani plants regularly prescribed by local practitioners to cure various ailments. Some of them have also been reported to have antioxidant activity [14-16]. These plants have been reported to show several activities, e.g. the tubers of *C. scariosus *are credited with astringent, diaphoretic, diuretic etc. properties; *R. damascena *flower buds are astringent and are used in cardiac troubles etc.; *V. wallichii *roots possess antiplasmodic properties and have a depressant effect on the central nervous system; and finely powdered *O. mascula *roots boiled with milk form a nutritious item of diet that is administered for diabetes, phthisis, chronic diarrhoea and dysentery [17]. Fresh leaf juice from the plant *C. icosandra *has been taken orally for toothache, whereas the seeds have been claimed to have anthelmintic properties. The bark of *H. anidysenterica *has astringent, antidysenteric, anthelmintic, stomachic, febrifugal etc. properties [18]. Gum obtained from *G. gummifera *is used internally in dyspepsia accompanied by flatulence and is also considered antispasmodic and carminative, antiseptic and stimulating. The roots of *A. pyrethrum *excite a remarkable flow of saliva and possess stimulating and rubefacient properties, whereas the leaves are used as carminatives, astringents etc. and the seeds are considered to be diuretic. *A. pindrow *leaves are carminative, astringent and antipyretic, and also used in asthma and bronchitis. A. *tenuifolius *seeds are considered to be diuretic [19]. These plants are edible and so considered safe [20]. The plants and their parts evaluated in this study are listed in Table 1. To date, few studies have been carried out to evaluate their antioxidant properties. Here we report our evaluation of their in vitro antioxidant potential, including the scavenging of DPPH^·^, ABTS^·+^, NO, ^·^OH, O_2_^.- ^and ONOO^-^, along with their activity in preventing oxidative DNA damage and cytotoxicity against U937 cells. To evaluate the mechanism of action of anti-oxidant properties of these plants, the total polyphenol and total flavonoid contents of all ten extracts were determined.

**Table 1:** Properties of Unani plants used in this study

Family	**Common name **^**1/2**^	Botanical name	Parts used	Uses	Class of compound	Name of compound
Capparidaceae	Dog mustard/Hurhur	*Cleome icosandra*	Seed^3^, leaves, flower	It is vata and kapha suppressant, a good pain reliever, also a good antibacterial and antiwormal, reduces pus formation in the wounds, helpful in convulsions, has a good effect on digestive tract and improves indigestion condition in the body, increases sweating in the body	Coumarino-lignans	Cleomiscosin A, C,
Rosaceae	Golap/Gulab	*Rosa damascena*	Petals^3^	Gulkhand made by the mixture of rose petals and white sugar in equal proportion act as the tonic and laxative, used as herbal tea in the treatment of cold and cough	Components of Essential oil Flavonol and their glycosides Flavonoid	Citronellol, Geraniol, Linalool etc. Glycosides of Kaempferol and Quercetin Quercetin, Kaempferol
Cyperaceae	Umbrella's edge/Nagarmotha	*Cyperus scariosus*	Root^3^	Intestinal disorders, astringent, diaphoretic, diuretic, desiccant, cordial, and stomachic properties, treatment of gonorrhea	Essential Oils	Some volatile compounds reported till date from the oil are atchoulanol, selina-4, etc.
Rubiaceae	Gummy gardenia/Dikamali	*Gardenia gummifera*	Resin^3^	Kapha skin disease, indigestion, worm infestation, diarrhea and infections, the resin has antiseptic property	Flavonoids, Flavone Seco-cycloartenol derivatives	Gardenin E Dikmaliartanes
Pinaceae	Himalayan fir/Dodimma	*Abies pindrow*	Bark^3^, leaves, trunk	Disorders with inflammatory system	Proanthocyanidins	Potential rich sources up to 5% of bark weight
Valerianaceae	Gilgiti valerian/Ganeshpawrobati	*Valeriana wallichii*	Roots^3^	Antisplasmodic, stimulant, calmative and stomachic, useful in diseases of eye and liver, used as a remedy for hysteria, hypochondriasis, nervous unrest and emotional arrest, also useful in clearing voice and acts as a stimulant in advance stage of fever and nervous disorder	Essential oil and volatile oil Iridous	0.3-1% volatile oil content Valtrate, didrovaltrate, acetovaltate, etc.
Apocynaceae	Tellycherry bark/Kurchi	*Holarrhena antidysenterica*	Bark^3^	The bark is used as an astringent, anthelmintic, antidontalgic, stomachic, febrifuge, antidropsical, diuretic, in piles, colic, dyspepsia, chest affections and as a remedy in diseases of the skin and spleen, use as a well-known drug for amoebic dysentery and other gastric disorders	Poly-phenolics Plant sterols	β-sitosterol
Asteraceae	Pellitory/Akarkara	*Anacyclus pyrethrum*	Root^3^	Stimulant, sialogogue, and rubefacient properties	Glycosides Phenolic acids Lignans	Flavone glycosides Chlorogenic acid Sesamin
Orchidaceae	Orchid/Salebpanja	*Orchis mascula*	Flower, tuber^3^	Tonic, aphrodisiac, yield a lot of mucilage with water and form a jelly that is supposed to be nutritious and useful in diarrhea, dysentery, and chronic fever	Bitter principle and a volatile oil	
Asphodelaceae	Onion weed/Jangli pyaz	*Asphodelus tenuifolius*	Bulb^3^, seed	As diuretic and on inflammation	Flavonoid	Luteolin and its glycosides

^1^English name ^2^Hindi name ^3^Plant part used in this study

## Methods

### Plant materials and extraction procedure

Plants were collected from, and authenticated by, a Unani medical practitioner in Kolkata, India who regularly prescribes these materials. The different plant parts were shade-dried at room temperature (25°C) with occasional turning of the plants upside down for 5-7 days, and then ground to coarse powder with a mechanical grinder. The powdered plant materials (2 g) were extracted with 50 ml of aqueous methanol (50:50) for three consecutive days with intermittent stirring (1 h stirring at every 12 h interval) using magnetic stirrer until the extracts were light colored. The combined extracts were filtered and evaporated under reduced pressure in a rotary vacuum evaporator (Eyela NVC-2100--Rotary Evaporator, water bath temperature maintained at 40°C and 356 mm Hg, Eyela NCB-1200 Chiller unit temperature maintained at 7.5°C). The aqueous layer was lyophilized (at -45°C) and the dry powder was stored at -20°C for future use.

### Reagents used

2, 2-Diphenyl-1-picrylhydrazyl (DPPH), thiobarbituric acid (TBA), Folin--Ciocalteu's phenol reagent, butylated hydroxytoluene, agarose and ethidium bromide were purchased from Sigma-Aldrich, USA. 2,2'-Azinobis-(3-ethyl-benzothiazoline-6-sulfonic acid) (ABTS), potassium persulphate, aluminium chloride, iron (III) chloride, and iron (II) sulphate were obtained from MP Biomedicals, USA. 2-Deoxy-D-ribose and ascorbic acid were procured from Himedia Laboratories Pvt. Ltd., Mumbai, India. The QIAprep Spin Miniprep Kit was purchased from Qiagen, Germany. All other chemicals and reagents used were of analytical grade.

### Analytical studies

For all the analytical studies, absorbance was measured using a Shimadzu UV-Visible Pharmaspec 1700 spectrophotometer.

### Determination of total phenolic content (TPC)

The total phenolic contents of the 50% methanolic plant extracts were determined with gallic acid as a positive standard [21]. Aliquots of test samples (100 μl) were mixed with 2 ml 2% Na_2_CO_3 _and incubated at 25°C for 2 min. After incubation, 1:1 (v/v) Folin-Ciocalteu's phenol reagent was added and the contents were mixed vigorously. The mixture was allowed to stand at 25°C for 30 min and the absorbance was measured at 720 nm. The same procedure was repeated with all standard gallic acid solutions and a standard curve was obtained. The total polyphenolic contents of the extracts were expressed in terms of gallic acid equivalents (GAE) of the plant sample.

### Determination of total flavonoid content (TFC)

The total flavonoid content was determined using quercetin as a positive standard and expressed in terms of quercetin equivalents (QEE) in mg/g plant sample [22]. NaNO_2 _(150 μl, 5% w/v) was added to tubes containing plant extracts in 2.5 ml distilled water. The contents were mixed thoroughly and allowed to stand for 5 min at ambient temperature, then 1.5 ml of 10% (w/v) AlCl_3 _were added and the mixture was allowed to stand for another 6 min. The solution was immediately mixed after addition of 1 ml 1 M NaOH. After 10 min, the absorbance was measured at 510 nm.

### Determination of total ascorbic acid (ASC)

ASC of plant extracts were determined according to Roe and Kuether [23] after brief modification. Blanks, standards and samples were prepared in triplicate to measure ASC. Ascorbic acid (AA) standards (0-10 mM) or samples were precipitated with 10% trichloroacetic acid followed by centrifugation. In 500 μL of supernatant, 100 μL of DTC reagent (2,4-dinitrophenylhydrazine 3%, thiourea 0.4%, and copper sulfate 0.05%) prepared in 9N sulfuric acid, was mixed and incubated at 37°C for 3 h. After the addition of 750 μL of 65% (v/v) sulfuric acid, the absorbance was recorded at 520 nm. A standard curve was prepared with AA standards, and ASC was expressed as mg AA/g of plant sample.

### Determination of free radical scavenging activity

The radical scavenging activities of the plant extracts in the range 0-200 μg/ml were evaluated using DPPH^·^. Stock solutions of plant extracts were prepared at a concentration of 10 mg/ml and a freshly-prepared DPPH solution (100 mM) was used as described previously [7].

Scavenging of ABTS^·+ ^was assayed to assess the antioxidant capacities of the 50% methanolic plant extracts according to the method of Re et al. [24]. The ABTS stock solution was prepared by reacting ABTS (7 mM) and potassium persulphate (2.45 mM) and allowing the mixture to stand for at least 16 h to generate ABTS^·+ ^free radicals. The working solution was prepared by diluting the stock solution with methanol such that its absorbance reached 0.7 ± 0.02 at 734 nm (*A*_Control_). For the assays, 1 ml of ABTS^·+ ^working solution was mixed with 10 μl extracts of different concentrations (0-100 μg/ml). Their absorbance (*A*_Sample_) was noted at 734 nm exactly 6 min after the reaction mixture was prepared. In both assays, quercetin was used as positive control. The control reaction contained no test sample. The percentage radical scavenging activity (% RSC) was calculated using the formula:

$$\%\text{ RSC}=[({\text{A}}_{\text{Control}}-{\text{A}}_{\text{Sample}})/{\text{A}}_{\text{Control}}]\times 100\%$$

### Determination of hydroxyl radical scavenging activity

The ^·^OH scavenging assay was performed as standardized before [8]. The reaction mixture consisted of different concentrations (0-100 μg/ml) of plant extract, 3.6 mM deoxyribose, 0.1 mM EDTA, 0.1 mM L-ascorbic acid, 1 mM H_2_O_2 _and 0.1 mM FeCl_3_.6H_2_O, and the volume was made up to 500 μl with 25 mM phosphate buffer, pH 7.4. This mixture was incubated for 1 h at 37°C, 500 μl of 1% TBA and 500 μl of 1% TCA were added, and the mixture was heated in a boiling water-bath for 15 min and then cooled. The absorbance was measured at 532 nm. The control reaction contained no test sample, and quercetin (20 μg/ml) was used as a standard. Percentage RSC was calculated as described above.

### Determination of peroxynitrite scavenging activity

Peroxynitrite was synthesized by the method of Beckman et al. 1994 [25]. Briefly, an acidic solution of 0.7 M H_2_O_2 _was mixed with an equal volume of 0.6 M potassium nitrite in an ice bath and an equal volume of ice cold 1.2 M NaOH was added. Granular MnO_2 _prewashed with 1.2 M NaOH was used to remove excess H_2_O_2 _and the reaction mixture was left at -20°C. The concentration of peroxynitrite generated was measured spectrophotometrically at 302 nm (ε = 1670 M^-1 ^cm^-1^).

Peroxynitrite scavenging activity was measured according to Hazra et al. 2010 [26]. The reaction mixture consisted of 0.1 mM DTPA, 90 mM NaCl, 5 mM KCl, 12.5 μM Evans Blue, plant extracts at various doses ranging from 0-300 μg/ml, and 1 mM peroxynitrite adjusted to a final volume of 1 ml with 50 mM phosphate buffer (pH 7.4). The reaction mixture was incubated at 25°C for 30 min and the absorbance was measured at 611 nm. The percentage peroxynitrite scavenging activity was calculated by comparing the results of the test and blank samples; gallic acid served as the reference compound. All tests were conducted six times. The IC_50 _values of the extracts were calculated by regression analysis.

### Determination of non-enzymatic superoxide radical scavenging activity

Superoxide radical was generated *in vitro *by a non-enzymatic method involving the nicotinamide adenine dinucleotide-nitro blue tetrazolium-phenazine methosulphate (NADH-NBT-PMS) system following the procedure of Nishikimi et al. [27]. NBT (150 μM in 0.02 M Tris buffer, pH 8.0) was added to 1 ml of NADH solution (50 μM of NADH in 0.02 M Tris buffer, pH 8.0) in the presence of various concentrations (0-50 μg/ml) of extracts. The reactions were initiated by adding PMS (15 μM) and the absorbance was at 560 nm was measured exactly 1 min later. Results were recorded as percentage inhibition. Quercetin at various concentrations was used as standard. All tests were performed six times.

### Nitric oxide scavenging activity: concentration dependence

The scavenging activity against nitric oxide was assayed by the method of Marcocci et al. [28]. Sodium nitroprusside (0.5 ml, 5 mM in 20 mM phosphate buffer, pH 7.4, previously bubbled with argon) was added to tubes containing 0.5 ml of different plant extracts of various concentrations (0-300 μg/ml) and incubated at 25°C for 150 min. At the end of the incubation, 1 ml of Griess reagent (equal volumes of 2% w/v sulphanilamide in 5% phosphoric acid and 0.2% w/v naphthylethylenediamine dihydrochloride) was added to each sample and the absorbance was measured at 546 nm against control samples (extracts incubated with only 20 mM phosphate buffer, pH 7.4) and referred to the absorbance of standard solutions of sodium nitrite treated in the same way with Griess reagent. Results were recorded as percentage nitrite formed. Quercetin at various concentrations was used as standard.

### Prevention of oxidative DNA damage

This was determined as described previously [8]. Plasmid DNA was isolated using a QIAprep Spin Miniprep Kit according to the manufacturer's instructions. Plasmid pBluescript II SK (-) (250 ng) was treated with FeSO_4_, H_2_O_2 _and phosphate buffer (pH 7.4) in final concentrations of 0.5 mM, 25 mM and 50 mM, respectively, and test extracts at different concentrations (0-2 μg/ml). The total reaction volume was set to 12 μl and the mixture was incubated at 37°C for 1 h. After the incubation, the extent of DNA damage and the preventive effect of the test samples were analyzed on 1% agarose gels at 70 V at room temperature. Quercetin (1 mM) was used as positive control.

Gels were scanned on a Gel documentation system (GelDoc-XR, Bio-Rad, Hercules, CA, USA). Bands were quantified using discovery series Quantity One 1-D analysis software (Bio-Rad).

### In vitro cytotoxicity activity (MTT assay)

The cytotoxicity of the plant extracts against the U937 cells was determined using the MTT (thiazolyl blue tetrazolium bromide) assay adapted from Kim et al. [29]. Cells were seeded into 96-well plates at 5,000-10,000 cells/well and treated with different concentrations of the plant extracts. After 48 h, MTT was added to each well and the formazan crystals were dissolved in DMSO. The absorbance was measured at 570 nm using a microplate ELISA reader. All experiments were performed in eight replicates. Percentage cell survival was calculated using the following formula:

$$\%\text{ cell survival}=[({\text{A}}_{\text{t}}-{\text{A}}_{\text{b}})/({\text{A}}_{\text{c}}-{\text{A}}_{\text{b}})]\times 100$$

Where A_t _= absorbance of test sample; A_b_= absorbance of blank (medium); A_c_= absorbance of control (cells).

### Statistical analysis

All data were expressed as mean ± SD. Statistical analyses were performed using Microsoft Excel. The IC_50 _values were calculated by regression analysis. Values with *p *< 0.05 were considered statistically significant. The IC50 values were compared by paired t test (two-sided).

## Results

### Total phenolic, flavonoid and ascorbic acid contents

Ten selected plants regularly prescribed in Unini system of medicine was investigated here (Table 1). 50% methanolic extracts of different parts of the were determined TPC, TFC and ASC were expressed as mg GAE/g extract, mg QEE/g extract and mg AA/g extract, respectively (Table 2).

**Table 2:** Total phenolic, flavonoid and ascorbic acid contents of plant extracts

Plant name	Total phenolic content **mg GAE/g plant extract**^**1**^	Total flavonoid content **mg QEE/g plant extract**^**1**^	Total ascorbic acid content **mg AA/g plant extract**^**1**^
*Cleome icosandra*	166.13 ± 0.56	172.23 ± 0.08	0.98 ± 0.218
*Rosa damascena*	142.23 ± 0.09	151.32 ± 0.51	0.82 ± 0.092
*Cyperus scariosus*	128.83 ± 0.32	118.93 ± 0.23	0.39 ± 0.017
*Gardenia gummifera*	82.72 ± 0.03	87.32 ± 0.13	0.49 ± 0.029
*Abies pindrow*	76.82 ± 0.13	63.82 ± 10.71	0.47 ± 0.079
*Valeriana wallichii*	72.13 ± 0.51	74.32 ± 0.21	0.55 ± 1.012
*Holarrhena antidysenterica*	69.12 ± 0.35	60.42 ± 0.34	0.42 ± 0.077
*Anacyclus pyrethrum*	62.89 ± 0.43	38.89 ± 0.52	0.37 ± 0.12
*Orchis mascula*	12.52 ± 0.57	12.11 ± 1.20	0.33 ± 0.073
*Asphodelus tenuiofolius*	15.74 ± 0.98	11.98 ± 0.74	0.14 ± 0.091

^1^Results are mean ± SD from three sets of independent experiments, each set in triplicate

The mean values of total phenols ranged from 62.89 ± 0.43 to 166.13 ± 0.56 mg GAE/g, flavonoids from 38.89 ± 0.52 to 172.23 ± 0.08 mg QEE/g extract and ASC from 0.14 ± 0.091 to 0.98 ± 0.218 AA/g extract. The highest TPC was observed in *C. icosandra *(166.13 ± 0.56 mg GAE/g extract), followed by *R. damascena *(142.23 ± 0.09 mg GAE/g extract) and *C. scariosus *(128.83 ± 0.32 mg GAE/g extract). For TFC, *C. icosandra *(172.23 ± 0.08 mg QEE/g extract) showed the highest content, also followed by *R. damascena *(151.32 ± 0.51 mg QEE/g extract) and *C. scariosus *(118.93 ± 0.23 mg QEE/g extract). The ASC contents were 0.98 ± 0.21, 0.82 ± 0.092 and 0.39 ± 0.017 mg AA/g extract in *C. icosandra, R. damascena *and *C. scariosus*, respectively followed by other extracts.

### DPPH^· ^scavenging activity

The free radical scavenging activities of the extracts, as measured by the ability to scavenge DPPH free radicals, were compared with quercetin; the lower the IC_50_, the stronger the scavenging activity (Table 3). The maximum % inhibition of the following extracts was noted like 85% in *C. icosandra *at 12.37 ± 1.09 μg/ml, 83% in *R. damascena *at 17.19 ± 0.23 μg/ml and 80.52% in *C. scariosus *at 17.71 ± 0.71 μg/ml followed by other extracts.

**Table 3:** IC50 values of plant extracts (μg/ml)

Plant Name	**DPPH**^**·**^	**ABTS**^**·+**^	^**·**^**OH**	NO	**O**_**2**_^**.-**^	**ONOO**^**-**^
*Cleome icosandra*	7.28 ± 0.37**	2.54 ± 0.04***	20.13 ± 0.01***	152.23 ± 3.51***	30.96 ± 0.98***	532.85 ± 15.93*
*Rosa damascena*	10.36 ± 0.02***	3.57 ± 0.11**	23.01 ± 0.03**	273.18 ± 3.52***	42.10 ± 0.82^NS^	637.57 ± 52.93**
*Cyperus scariosus*	11.10 ± 0.37**	6.27 ± 0.44**	18.23 ± 0.038***	240.31 ± 4.28***	28.85 ± 0.23***	590.23 ± 2.37**
*Gardenia gummifera*	82.33 ± 0.31***	11.62 ± 0.21**	34.33 ± 0.07***	--	45.39 ± 0.87***	890.32 ± 52.23***
*Abies pindrow*	84.23 ± 1.50**	19.10 ± 0.21***	31.43 ± 0.07***	286.59 ± 3.89***	74.54 ± 9.28***	987.42 ± 17.4***
*Valeriana wallichii*	86.61 ± 0.89**	21.26 ± 0.18***	37.92 ± 0.07***	--	78.35 ± 0.57***	943.12 ± 27.82***
*Holarrhena antidysenterica*	98.84 ± 0.31***	29.92 ± 0.25***	29.23 ± 0.01**	211.34 ± 2.12***	83.49 ± 0.59***	880.51 ± 9.99***
*Anacyclus pyrethrum*	467.10 ± 0.27***	31.76 ± 0.27***	41.22 ± 0.04***	--	83.49 ± 0.59***	1.137 ± 0.003^1^***
*Orchis mascula*	1.098 ± 0.009^1^***	--	47.82 ± 0.20**	--	537.87 ± 93.12**	3.114 ± 0.09^1^***
*Asphodelus tenuifolius*	2.006 ± 0.002^1^***	156.94 ± 5.28***	50.13 ± 0.04***	--	425.92 ± 78.12***	3.390 ± 0.031^1^***
Reference Compound						
Quercetin	3.21 ± 0.11	1.34 ± 0.08	7.42 ± 0.32	18.23 ± 0.42	41.98 ± 0.95	
Gallic acid				--		820.12 ± 27.34

Results are mean ± SD (n = 3), each set in triplicate

Units of IC_50_: (mg/ml)^1^

* p < 0.05; ** p < 0.01;*** p < 0.001; NS = Non significant

### ABTS^·+ ^scavenging activity

The IC_50 _values of the plant extracts were also determined for ABTS^·+ ^(Table 3). Significant activity was noted with *C. icosandra, R. damascena *and *C. scariosus *with 98.23% inhibition at 6.98 ± 0.07 μg/ml, 91.83% inhibition at 8.42 ± 0.13 μg/ml and 72% inhibition at 8.32 ± 0.09 μg/ml, respectively.

### ^·^OH scavenging activity

The ^·^OH scavenging potentials manifested by the different plant extracts were also evaluated by decreased formation of the chromogen in the Fenton reaction. The ^·^OH scavenging activities of the 50% methanolic extracts correlated with protection against DNA damage, as shown in Table 3. Best scavenging activity was noted with *C. icosandra, R. damascena *and *C. scariosus *which showed 67.08% inhibition at 34.54 ± 0.92 μg/ml, 69.7% inhibition at 23.48 ± 0.85 μg/ml and 67.2% inhibition at 29.33 ± 0.43 μg/ml, respectively.

### Peroxynitrite scavenging activity

In all the extracts tested, peroxynitrite-scavenging activity was concentration dependent. The scavenging activities was, 72% inhibition at 766.08 ± 12.23 μg/ml, 78% inhibition at 993.72 ± 52.34 μg/ml and 69% inhibition at 814.2 ± 37.89 μg/ml for *C. icosandra*, *R. damascena and C. scariosus *respectively. Hence these three extracts have superior activity than that of gallic acid standard. Howerver, IC_50 _values of *H. antidysenterica *(880 ± 9.99 μg/ml) and *G. gummifera *(890 ± 52.23 μg/ml) were comparable to the standard (i.e. 820.12 ± 47.2 μg/ml) in peroxynitrite scavenging potential (Table 3).

### Superoxide scavenging activity

As is evident from Table 3, the extracts of *C. icosandra *(73% inhibition at 45.20 ± 8.25 μg/ml), *R. damascena *(81% inhibition at 68.20 ± 7.23 μg/ml) and *C. scariosus *(78.23% inhibition at 45.23 ± 0.37 μg/ml) also caused considerable scavenging of superoxide anion in comparison to the reference compound quercetin. The IC_50 _values for the superoxide scavenging activities of extracts and the reference standard are shown in Table 3. As evident from results, *C. scariosus *(28.85 ± 0.23 μg/ml) was able to quench superoxide radicals more effectively than the reference compound quercetin (41.98 ± 0.95 μg/ml).

### Nitric oxide scavenging activity

*C. icosandra *showed significant nitric oxide scavenging activity than that of other plant extracts having 69% inhibition at 210.07 ± 18.27 μg/ml. However modest scavenging activity was also noted with *R. damascena *(73.9% at 398.84 ± 52.1 μg/ml) and *C. scariosus *(72.24% at 350.85 ± 12.3 μg/ml) respectively. IC_50 _values were presented in Table 3 along with reference compound quercetin (at 18.23 ± 0.42 μg/ml).

### Prevention of oxidative DNA damage by plant extracts

**Figure 1:**
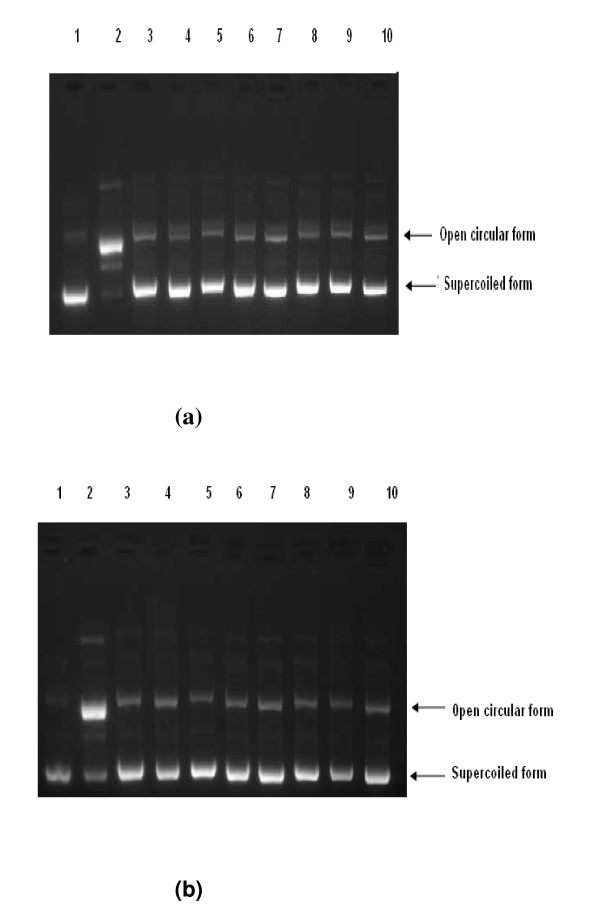
**Electrophoresis patterns of pBluescript II SK (--) DNA breaks by ^·^OH generated from the Fenton reaction and prevented by different plant extracts.** (a) Lane 1: untreated control DNA (250 ng); lane 2: FeSO_4 _(0.5 mM) + H_2_O_2 _(25 mM) + DNA (250 ng); lane 3: only H_2_O_2 _(25 mM) + DNA (250 ng); lane 4: only FeSO_4 _(0.5 mM) + DNA (250 ng); lanes 5--10: FeSO_4 _(0.5 mM) + H_2_O_2 _(25 mM) + DNA (250 ng) in the presence of quercetin (1 mM), *Gardenia gummifera *(0.53 μg/ml), *Abies pindrow *(0.29 μg/ml), *Asphodelus tenuifolius *(1.85 μg/ml), *Anacyclus pyrethrum *(1.52 μg/ml) and *Orchis mascula *(1.60 μg/ml), respectively (n = 3). (b)  Lane 1: untreated control DNA (250 ng); lane 2: FeSO_4 _(0.5 mM) + H_2_O_2 _(25 mM) + DNA (250 ng); lane 3: only H_2_O_2 _(25 mM) + DNA (250 ng); lane 4: only FeSO_4 _(0.5 mM) + DNA (250 ng); lanes 5--10: FeSO_4 _(0.5 mM) + H_2_O_2 _(25 mM) + DNA (250 ng) in the presence of quercetin (1 mM), *Holarrhena antidysenterica *(0.28 μg/ml), *Valeriana wallichii *(1 μg/ml), *Rosa damascena *(0.20 μg/ml), *Cleome icosandra *(0.16 μg/ml) and *Cyperus scariosus *(0.13 μg/ml), respectively (n = 3).

**Figure 2:**
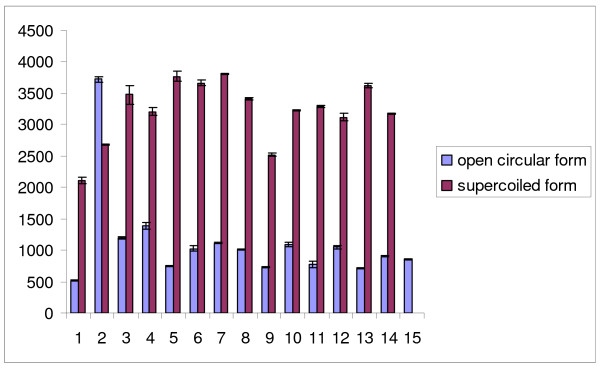
**Densitometric analysis of open circular and supercoiled DNA damage induced by ^·^OH generated from the Fenton reaction in the presence of plant extracts**. Lane 1: untreated control DNA (250 ng); lane 2: FeSO_4 _(0.5 mM) + H_2_O_2 _(25 mM) + DNA (250 ng); lane 3: only H_2_O_2 _(25 mM) + DNA (250 ng); lane 4: only FeSO_4 _(0.5 mM) + DNA (250 ng); lanes 5--15: FeSO_4 _(0.5 mM) + H_2_O_2 _(25 mM) + DNA (250 ng) in the presence of quercetin (1 mM), *Gardenia gummifera *(0.528 μg/ml), *Abies pindrow *(0.29 μg/ml), *Asphodelus tenuifolius *(1.85 μg/ml), *Anacyclus pyrethrum *(1.52 μg/ml), *Orchis mascula *(1.60 μg/ml) *Holarrhena antidysenterica *(0.28 μg/ml), *Valeriana wallichii *(1 μg/ml), *Rosa damascena *(0.2 μg/ml), *Cleome icosandra *(0.16 μg/ml) and *Cyperus scariosus *(0.128 μg/ml) respectively. Values represent mean ± SD (n = 3). The differences were considered statistically significant if *p *< 0.05.

To assess the prevention of oxidative DNA damage by the plant extracts further, the preventive effects were evaluated over Fenton-induced damage of pBluescript II SK (-) supercoiled DNA maintained in *E. coli *XL-1 Blue strain. Control pBS DNA showed two bands, one of open circular DNA, which was hardly visible, and one of supercoiled DNA. Combined treatment with FeSO_4 _and H_2_O_2 _in the absence of plant extract led to the formation of open circular DNA by strand scission of the supercoiled DNA, whereas the plant extracts at different concentrations showing optimum activity prevented this strand scission. The maximum prevention of DNA damage was shown by *C. scariosus *at 0.13 μg/ml whereas *C. icosandra *showed the same activity at 0.16 μg/ml. *R. damascena, A. pindrow, G. gummifera, O. mascula, A. pyrethrum, A. tenuifolius, H. antidysenterica *and *V. wallichii *showed the same preventive activity at 0.2 μg/ml, 0.22 μg/ml, 0.53 μg/ml, 1.60 μg/ml, 1.52 μg/ml, 1.85 μg/ml, 0.1 μg/ml and 1 μg/ml, respectively. Among these plant extracts, *C. scariosus, C. icosandra, R. damascena *and *H. antidysenterica *provided the most effective prevention of DNA damage, as shown in Figure 1. Densitometric analysis confirmed the experimental data (Figure 2).

### Correlation between the TPC or TFC with the antioxidant activity

**Figure 3:**
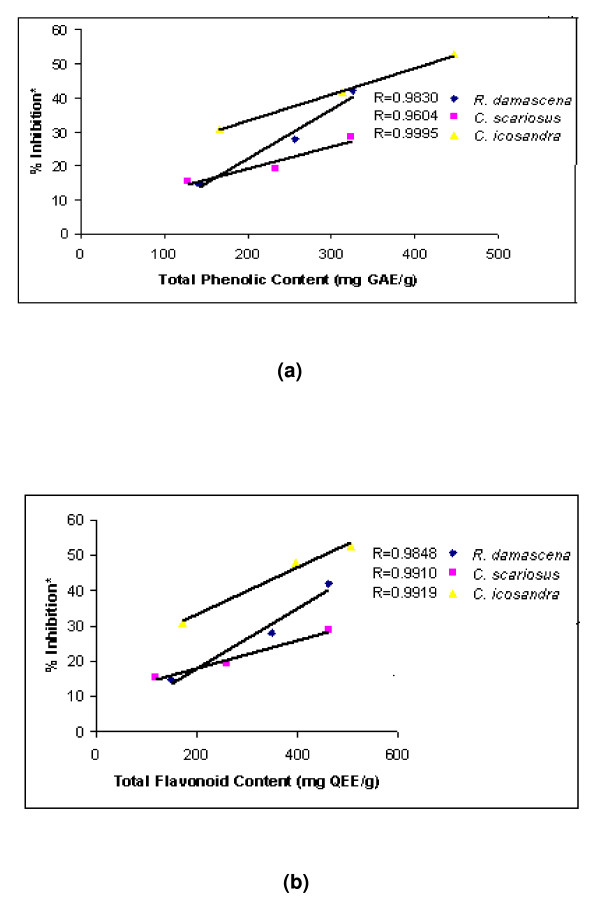
**Correlation of % inhibition with Phenolic and Flavonoid contents**. The relationship between (a) total phenolics content or (b) total flavonoid content in *R. damascena*, *C. scariosus, C. icosandra *and their antioxidant activity. The correlation analysis was described as linear correlation coefficient (R). The differences were considered statistically significant if *p *< 0.05.% Inhibition* = free radical scavenging activity as determined by ABTS assay.

Correlation of phenolics content and antioxidant activity of three plant extracts with superior antioxidant activity was determined. Results showed that the correlation coefficients of total phenolics and flavonoid contents of *C. icosandra *were greater than 0.9 (*R *= 0.9995; *R *= 0.9919 respectively), the same of *R. damascena R *= 0.9830; *R *= 0.9848) and *C. scariosus *(*R *= 0.9604; *R *= 0.9910) was comparative as shown in Figure 3. This signified that the oxidative DNA damage preventive activity as well as antioxidant potential of these three effective plant extracts could be strictly correlated with their total phenolics and flavonoid contents.

### Cytotoxicity

The cytotoxic activities of the extracts of *R. damascena, C. scariosus *and *C. icosandra *at different concentrations (0.2 and 2 μg/ml) were determined against U937 cells (Table 4). These extracts showed no cytotoxicity against U937 cells in comparison to doxorubicin.

**Table 4:** Cytotoxic activity of three plants at two different concentrations

Time	*Rosa damascena*		*Cyperus scariosus*		*Cleome icosandra*		Doxorubicin 25 ng/ml
	0.2 μg/ml	2 μg/ml	0.2 μg/ml	2 μg/ml	0.2 μg/ml	2 μg/ml	
00 h	100%	100%	100%	100%	100%	100%	100%
24 h	97.46%	95.77%	96.37%	96.47%	99.72%	96.81%	42%
48 h	97.58%	96.95%	96.20%	96.06%	99.39%	97.03%	0%
72 h	97.37%	97.08%	96.59%	96.11%	98.86%	96.75%	0%

^1^Results are mean from three sets of experiments, each set in five replicate.

## Discussion

As a part of a concerted effort to develop herbal antioxidants from natural sources, we investigated several plants regularly prescribed in the Unani system of medicine against various human ailments. For initial free radical screening, DPPH assay followed by an ABTS assay was used which showed significant activity in *C. icosandra, R. damascena *and *C. scariosus*. To evaluate this potential more specifically, extracts were checked for ^·^OH scavenging and the highest activity was noted with *C. icosandra, R. damascena *and *C. scariosus*, corroborating the previous assay. Significant NO scavenging was noted with *C. icosandra*, followed sequentially by *H. antidysenterica, C. scariosus *and *R. damascena*. Peroxynitrite scavenging by *C. icosandra*, *C. scariosus*, and *R. damascena *was significantly greater than by the reference compound, whereas *H. antidysenterica *and *G. gummifera *showed similar activity to the standard compound. O_2_^.- ^scavenging activity was also significant in the extracts of *C. icosandra*, *C. scariosus *and *R. damascena*. Taken together, these findings indicate that *C. icosandra *extract is a potential candidate for free radical scavenging followed by *R. damascena *and *C. scariosus*.

Phytochemical analysis revealed significant total phenolic and flavonoid contents in the extracts of these same three plants, *C. icosandra, R. damascena *and *C. scariosus*, and these correlated with their potential radical scavenging activities. Though the ASC of these three effective extracts were insignificant, however that of *C. icosandra *and *R. damascena *was little higher than of *C. scariosus*, indicating that the antioxidant potential of *C. scariosus *arises from its total phenolic and flavonoid contents. Other plant extracts are also reported to contain polyphenolic compounds and their antioxidant activities may be related to this [30].

Flavonoids are polyphenols naturally present in nearly all plant materials [30]. Phenolic compounds are effective hydrogen donors, and this makes them good antioxidants [31]. Flavonoids are a class of compounds that remain of great scientific and therapeutic interest, and their antioxidant activity has attracted most attention. Their high antioxidant potential is attributable to their capacity to scavenge harmful ROS and other free radicals that originate from various cellular activities and lead to oxidative stress [32]. Plant-derived polyphenolic flavonoids are well known to exhibit antioxidant activity through a variety of mechanisms including scavenging of ROS, inhibiting lipid peroxidation and chelating metal ions [33]. Hence their mechanism of action is multiple; it includes the inhibition of enzymes involved in ROS generation, chelating of trace metals such as free iron and copper, and the ability to reduce highly oxidizing free radicals by hydrogen donation, thus protecting us from serious diseases such as heart attacks, strokes and even cancers. In addition, ascorbic acid acting as a chain-breaking antioxidant impairs the formation of free radicals during the biosynthesis of intracellular and extracellular substances throughout the body, including collagen, bone matrix and tooth dentine [34].

Previous studies have reported that the seeds of *C. icosandra *contain coumarino-lignans such as cleomiscosin A, B, C and D, of which A and C are reported to be antioxidants [16,35]. Collectively, these observations indicate that the free radical scavenging potential of *C. icosandra *seeds and protection they confer against oxidative DNA damage may be attributed to their phytochemical composition. Rose essential oil is widely used in perfumery and the cosmetic industry. In addition to its perfuming effects, it is reported to possess a wide range of biochemical activities. Petals of *R. damascena *contain flavonol aglycons like kaempferol, quercetin and its glycosides such as kaempferol glycosides, quercitrin etc., citronellol and geraniol as the major components of its essential oil as well as tocopherol and carotene [19,36,37]. Potential antioxidant activity of rose petals may be attributed for their diversified phytochemical contents, which are consistent with earlier reports [36-38]. *C. scariosus *roots contain compounds such as patchoulanol, isopatchoulenone, etc. as major components of its essential oil [17]. Wei and Shibamoto [39] studied the antioxidant activities of major essential oils from several plants and reported that myristicin from parsley seeds, patchouli alcohol from patchouli, and citronellol from roses showed high antioxidant activities, which can be related to our study.

The Fenton reaction is a major physiological source of ^·^OH, which is produced near DNA molecules in the presence of transition metal ions such as iron and copper [40]. As previous reports suggest, polyphenol-rich diets may decrease the risk of chronic diseases by reducing oxidative stress [41]. The Fenton reaction is prevented by hydroxyl radical-scavenging flavonoids [42]. Here, the capacities of all ten plant extracts to protect against oxidative DNA damage were checked against DNA strand scission by ^·^OH generated in Fenton reactions on pBluescript II SK (--) DNA. We conclude that a significant contributor to DNA damage prevention is the scavenging of ^·^OH by the extracts of *C. scariosus, C. icosandra, R. damascena *and *H. antidysenterica *at 0.13 μg/ml, 0.16 μg/ml, 0.2 μg/ml and 0.28 μg/ml, respectively; this was corroborated by densitometric analysis.

The three effective extracts, viz. *C. icosandra, R. damascena *and *C. scariosus*, were not cytotoxic in comparison to doxorubicin, and this appears consistent with their long history of use in the Unani system of medicine.

## Conclusions

Unani plants that are reported to have significant activity against several human ailments showed superior antioxidant activity as evidenced by the scavenging of the free radicals DPPH^·^, ABTS^·+^, NO, ^·^OH, O_2_^.- ^and ONOO^-^. Of the ten 50% methanolic plant extracts tested, three - namely *C. icosandra, R. damascena *and *C. scariosus *- showed potentially significantly capacity to prevent oxidative DNA damage and radical scavenging activity. The *C. icosandra, R. damascena *and *C. scariosus *extracts were not cytotoxic against U937 cells. To gain further insight into the basis of their antioxidant properties, TPC, TFC and ASC were determined. All three extracts showed significantly high TPC and TFC contents, which contribute to their antioxidant activities. In conclusion, these routinely used plants can be explored further as potential sources of natural antioxidants.

## Competing interests

The authors declare that they have no competing interests.

## Authors' contributions

MDK carried out the experimental work, analyzed and interpreted the data and drafted the manuscript. DB made a substantial contribution to the conception and design of the study. AB contributed partially to the design of the study. SC supervised the work and revised the manuscript critically for important intellectual content. The authors have all read and approved the final manuscript.

## Pre-publication history

The pre-publication history for this paper can be accessed here:

http://www.biomedcentral.com/1472-6882/10/77/prepub
